# An intrinsically disordered region of methyl-CpG binding domain protein 2 (MBD2) recruits the histone deacetylase core of the NuRD complex

**DOI:** 10.1093/nar/gkv168

**Published:** 2015-03-09

**Authors:** Megha A. Desai, Heather D. Webb, Leander M. Sinanan, J. Neel Scarsdale, Ninad M. Walavalkar, Gordon D. Ginder, David C. Williams

**Affiliations:** 1 Department of Human and Molecular Genetics and Massey Cancer Center, Virginia Commonwealth University, Richmond, VA 23298, USA; 2 Department of Pathology and Massey Cancer Center, Virginia Commonwealth University, Richmond, VA 23298, USA; 3 Department of Pathology and Laboratory Medicine, University of North Carolina at Chapel Hill, Chapel Hill, NC 27599, USA; 4 Institute of Structural Biology and Drug Design, Center for the Study of Biological Complexity, and Massey Cancer Center, Virginia Commonwealth University, Richmond, VA 23298, USA; 5 Departments of Internal Medicine, Human and Molecular Genetics, and Microbiology and Immunology and Massey Cancer Center, Virginia Commonwealth University, Richmond, VA 23298, USA

## Abstract

The MBD2-NuRD (Nucleosome Remodeling and Deacetylase) complex is an epigenetic reader of DNA methylation that regulates genes involved in normal development and neoplastic diseases. To delineate the architecture and functional interactions of the MBD2-NuRD complex, we previously solved the structures of MBD2 bound to methylated DNA and a coiled-coil interaction between MBD2 and p66α that recruits the CHD4 nucleosome remodeling protein to the complex. The work presented here identifies novel structural and functional features of a previously uncharacterized domain of MBD2 (MBD2_IDR_). Biophysical analyses show that the MBD2_IDR_ is an intrinsically disordered region (IDR). However, despite this inherent disorder, MBD2_IDR_ increases the overall binding affinity of MBD2 for methylated DNA. MBD2_IDR_ also recruits the histone deacetylase core components (RbAp48, HDAC2 and MTA2) of NuRD through a critical contact region requiring two contiguous amino acid residues, Arg^286^ and Leu^287^. Mutating these residues abrogates interaction of MBD2 with the histone deacetylase core and impairs the ability of MBD2 to repress the methylated tumor suppressor gene *PRSS8* in MDA-MB-435 breast cancer cells. These findings expand our knowledge of the multi-dimensional interactions of the MBD2-NuRD complex that govern its function.

## INTRODUCTION

Epigenetic regulation comprises heritable changes in gene expression most commonly brought about by DNA methylation and histone modifications. The predominant form of DNA methylation in mammals involves addition of a methyl group to the C5 carbon of the cytosine residue in a cytosine-guanine dinucleotide (CpG) through the enzymatic activity of DNA methyl-transferases, DNMT1, DNMT3A and DNMT3B (1). Regions of high CpG density are often associated with gene promoters (2), which remain unmethylated except for a subset of tissue-specific genes involved in normal differentiation and development (3–6). Aberrant hypermethylation of tumor suppressor gene promoters is associated with oncogenesis in a wide array of tissues (7). The methyl-CpG binding domain (MBD) family proteins recognize this methylated mark and repress the associated genes by recruiting different co-repressor complexes. The MBD family of proteins include the first identified MeCP2 (8) and MBD1, MBD2, MBD3 and MBD4 (9). With the exception of mammalian MBD3, all MBD proteins bind to methylated DNA although with varying affinities (10–13).

MBD2 binds densely methylated CpG islands and represses transcription of the associated genes through recruitment of the Nucleosome Remodeling and Deacetylase (NuRD) co-repressor complex (14). The MBD2-NuRD co-repressor complex from both cell lines and primary cells has been characterized and is comprised of at least one copy each of the MTA1/2/3, HDAC1/2, RbAp46/48, p66α/β, and CHD3/4 and MBD2 proteins (5,14–16). Recent studies have evaluated the stoichiometry of protein interactions in the NuRD complex (15,16), however much remains to be explored about the nature and assembly of protein–protein interactions within this complex. Previous work in our laboratory identified MBD2 as a silencer of the chicken ρ-globin gene (5,17,18) as well as murine and human embryonic and fetal β-type globin genes in adult erythroid cells (4,18,19). In addition, MBD2 has been implicated in aberrant silencing of methylated tumor suppressor genes in carcinogenesis (20–26).

The MBD2 protein consists of an N-terminal glycine-arginine repeat region (GR), a methyl-binding domain (MBD) which binds *in vivo* to densely methylated DNA (27), an uncharacterized domain of MBD2 (MBD2_IDR_) and a coiled-coil domain. In previous work we showed that the C-terminal coiled-coil of MBD2 binds to the p66α component of NuRD, which contributes to the recruitment of CHD4 and gene silencing. Consistent with these findings, the p66α coiled-coil domain peptide can bind to native MBD2 in cells and relieve MBD2-mediated repression of target genes such as the embryonic and fetal β-type globin genes in adult erythroid cell culture systems (18). This proof-of-concept study underscored the biological significance of functional disruption of the MBD2-NuRD co-repressor complex and led us to pursue characterization of other MBD2 mediated interactions within the NuRD complex.

Intrinsically disordered proteins (IDPs) are a rapidly advancing area of research due to their importance in human biology. Although IDPs lack a stable three-dimensional structure under physiological conditions, they can serve as hubs of multi-protein interactions for diverse cellular functions including transcription regulation, chromatin remodeling and cell signaling because the intrinsic disorder permits transient, low affinity but high specificity protein–protein and nucleic acid-protein interactions (28). Among the MBD protein family members, the transcription repression domain of MBD1 (29) and 60% of full-length MeCP2 protein have been shown to be intrinsically disordered (30), even in the presence of their binding partners.

Having previously determined the structures of the MBD and coiled-coil domains of MBD2, we present here the unique structural and functional features of the previously uncharacterized MBD2_IDR_. We show that this region is intrinsically disordered in isolation and in the context of the full-length protein bound to DNA, we identify its role in modifying kinetics and affinity of DNA-binding, and map the critical sites needed for MBD2_IDR_ to recruit the histone deacetylase core complex within the context of the intact MBD2 protein in cells. We anticipate these results will facilitate efforts for further biochemical and structural characterization of the MBD2-NuRD complex and open up avenues to target co-repressor activities of MBD2-NuRD via disruption of MBD2_IDR_-mediated interactions with the NuRD complex.

## MATERIALS AND METHODS

### Protein expression and purification

**Figure 1.**
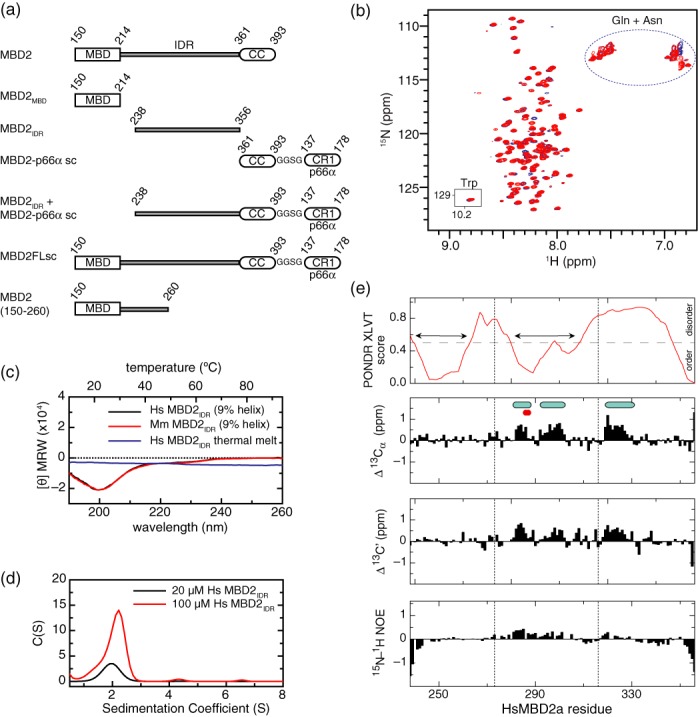
The MBD2_IDR_ of MBD2 is intrinsically disordered in isolation. (**a**) A schematic diagram shows the domain organization of the different constructs used in these studies including the methyl-cytosine binding domain (MBD), intrinsically disordered region (IDR), and coiled-coil (CC) domains as well as a single chain (sc) construct that includes a short GGSG linker and the coiled-coil domain (CR1) of p66α. (**b**) An overlay of ^15^N-HSQC spectra for the MBD2_IDR_ of *Hs*MBD2 (red) and *Mm*MBD2 (blue) shows a lack of chemical shift dispersion with highly degenerate Gln and Asn ^15^N-^1^H_2_ (blue circle) and Trp ^15^N-^1^Hϵ (inset) resonances. (**c**) CD spectra of the MBD2_IDR_ at 25°C for *Hs*MBD2 (black) and *Mm*MBD2 (red) show little evidence of secondary structure formation while a thermal melt for *Hs*MBD2 MBD2_IDR_ (blue) fails to reveal a cooperative transition characteristic of folded domains. (**d**) Fitting analytical ultracentrifugation sedimentation velocity data to a continuous size distribution (SEDFIT software (35)) indicates that the HsMBD2_IDR_ is largely monomeric at 20 μM (black) but does show evidence of slight oligomerization and aggregation at 100 μM (red) concentration. (**e**) PONDR® VLXT analysis of the MBD2_IDR_ is plotted in the upper panel which shows two sub-regions of low disorder propensity (indicated by horizontal double-headed arrows, amino acids 240–262 and 280–308) within the IDR. Secondary chemical shifts were calculated for the MBD2_IDR_ as described by Kjaergaard and Poulsen (41) and plotted for alpha (^13^C_α_) and carbonyl (^13^C’) resonances in the second and third panels. Regions with positive secondary chemical shifts consistent with α-helix formation are indicated with light blue ovals. Large red dots indicate the location of mutations that disrupt binding to the NuRD HDCC. Heteronuclear ^15^N-^1^H NOE for the MBD2_IDR_ are plotted in the lower panel showing that most of the domain is highly dynamic with values < 0.5. The vertical dotted lines in all four panels indicate the three different sub-regions used for mapping the interaction with the NuRD HDCC. The second sub-region contains a stretch of positive heteronuclear NOE values consistent with a propensity to form α-helices.

Various MBD2 constructs (Figure 1a) were cloned into a modified pET32a vector (31) including: the MBD2_IDR_ of human (amino acids 238–356) and mouse (amino acids 241–359) MBD2; a human single-chain coiled-coil domain construct (scMBD2-p66α) comprised of the MBD2 coiled-coil domain (amino acids 361–393), a short GGSG linker, and the p66α coiled-coil domain (amino acids 137–178); human MBD2_MBD_ construct comprised of the isolated MBD (amino acids 150–214), and a slightly longer construct that incorporates the MBD through the first twenty amino acids from the MBD2_IDR_ (amino acids 150–260), and a full-length human MBD2 single-chain construct (MBD2FLsc) comprised of MBD2 (amino acids 150–393) and the scMBD2-p66α. Note that the full-length construct does not incorporate the N-terminal GR repeat region unique to mammalian MBD2 proteins. The resulting plasmids were transformed into the Rosetta II (DE3) (Invitrogen) *E. coli* strain, grown in either Luria Bertani medium (unlabeled) or M9 minimal media (^13^C, ^15^N, ±^2^H labeled) and induced with 1mM isopropyl-β-d-thiogalactopyranoside at an A_600_ ∼ 0.8 for 2 or 4 h, respectively. Bacterial pellets from 1L of growth media were lysed with 30 ml of the B-PER reagent (Thermo Scientific) and the expressed fusion protein purified by nickel affinity chromatography. For analytical ultracentrifugation studies and DNA binding affinity studies, the fusion protein was further purified by gel filtration chromatography (Superdex-75 26/60, GE Healthcare). For circular dichroism (CD) and nuclear magnetic resonance (NMR) studies, the MBD2_IDR_ and scMBD2-p66α were separated from the thioredoxin fusion domain by thrombin cleavage at room temperature overnight, and further purified by gel filtration (Superdex-75 26/60, GE Healthcare) and ion exchange (MonoS 10/100, GE Healthcare) chromatography. The final protein concentration was determined by UV absorbance at 280 nm.

### MBD2FLsc:DNA complex preparation

Complementary 17 base pair oligonucleotides containing a central methylated CpG dinucleotide (5′-GAGGCGCT(**^me^C)**GGCGGCAG-3′) were purchased from Integrated DNA Technologies (IDT), annealed and purified by ion exchange chromatography (MonoQ 10/100, GE Healthcare) as described previously (32). The concentration of purified double-stranded oligonucleotide was determined by UV absorbance at 260 nm and the DNA added in 10% molar excess to purified uniform ^2^H, ^13^C, ^15^N-labeled MBD2FLsc. The resulting complex was concentrated and buffer exchanged into NMR buffer (see below).

### Surface plasmon resonance (SPR) analysis

3′ biotinylated forward oligonucleotide was purchased from IDT, annealed to the reverse strand and purified as described above. Protein and DNA samples were buffer exchanged into 10 mM HEPES pH 7.4, 50 mM NaCl, 3 mM MgCl_2_, 0.1 mM EDTA, 1 mM DTT. SPR analysis of binding was performed on a Biacore T100 (GE Healthcare) as described previously (10,33). The purified DNA was bound to a Sensor SA chip (10 ng/ul DNA, 10 ul/min flow rate, 100 s) until a final relative response of approximately 100 U. Kinetic binding analyses were carried out at a flow rate of 30 ul/min (10mM HEPES, 50mM NaCl, 3mM MgCl_2_, 0.1mM EDTA, 1mM DTT, 0.05% polysorbate 20, pH 7.4) and the data were fit using the manufacturer's software. As described previously, the rapid on and off-rates for the isolated MBD2_MBD_ precluded analysis of binding kinetics. Therefore, the binding constant for MBD2_MBD_ was determined by steady-analysis (10,33).

### Fluorescence polarization (FP) analysis

3′ FAM labeled reverse strand oligonucleotide was purchased from IDT, annealed to the forward strand and purified as described above. Protein and DNA samples were buffer exchanged into 10 mM HEPES pH 7.5, 3 mM MgCl2, 0.1 mM EDTA, 1 mM DTT, and 0.02% sodium azide with either 50 mM NaCl (low salt buffer) or 150 mM NaCl (high salt buffer). Serial dilutions of protein were added to 10 mM DNA and fluorescence polarization measured on a PHERAstar FS microplate reader (BMG Labtech). The dissociation constant (*K_D_*) was determined for each by fitting the observed polarization ([mP]) to a general equation for two state binding:

\begin{equation*} \begin{array}{*{20}c} {[mp] = [mP]_{{\rm DNA}} + } \\ {\frac{{\left( {[D] + K_D + [P] - \sqrt {\left( {[D] + K_D + [P]} \right)^2 - 4*[P]*[D]} } \right)}}{{2*[D]}}*} \\ {\left( {[mP]_{{\rm sat}} - [mP]_{{\rm DNA}} } \right),} \\ \end{array} \end{equation*}

in which [D] and [P] are the DNA and protein concentration and [mP]_DNA_ and [mP]_sat_ are the polarization for free and protein saturated DNA, respectively. The data were fit and plotted using Pro Fit software (QuantumSoft).

### Analytical ultracentrifugation

Protein was buffer exchanged into 20mM Tris pH 8.0, 150 mM NaCl and sedimentation velocity analyzed at 40 000 rpm, 20°C on a Beckman Optima XL-I analytical ultracentrifuge (Beckman Coulter Inc.) equipped with a four and eight-position AN-60Ti rotor. Sedimentation profiles were recorded for 20 μM and 100 μM samples using UV absorption (280 nm) and interference scanning optics. The sample partial specific volume, buffer density and viscosity were calculated with the SEDNTERP (34) software and the effective molecular weight determined by fitting the data to a continuous size distribution with the SEDFIT (35) software.

### Circular dichroism

The MBD2_IDR_ was buffer exchanged into 10 mM NaPO_4_, pH 6.5 at a final concentration of ∼100 μg/ml protein. CD spectra were collected from 185 to 260 nm (0.5 nm interval, 24 nm/min, 0.1 cm path length, 25°C) on a Chirascan™-plus CD spectrometer (Applied Photophysics). CD spectra were normalized to provide mean residue molar ellipticity ([θ] MRW) in degrees cm^−2^ dmol^−1^ residue^−1^. Helical content for each peptide was calculated as described previously (36,37). The temperature dependence of circular dichroism (thermal melt) was followed by measuring ellipticity at 222 nm at 1°C intervals over a temperature range of 10–94°C with a heating rate of 1°C /min.

### Nuclear magnetic resonance

Uniform ^13^C, ^15^N labeled protein was buffered exchanged into 10 mM NaPO_4_, pH 6.5, 0.02% sodium azide, 1mM dithiothreitol, and 10% ^2^H_2_O and concentrated to 0.5–1 mM. NMR spectra were collected on a Bruker Avance III 700 MHz instrument at 25°C, and data were processed and analyzed with NMRPipe (38) and CcpNmr (39), respectively. Standard double and triple resonance experiments (^15^N-HSQC, HNCO, HNCACB, CBCA(CO)NH, HBHA(CO)NH, HN(CA)NNH, HNCACO, CCH-TOCSY, (H)CC(CO)NH, ^15^N-NOESY-HSQC) were collected and the backbone and sidechain H_α/β_ resonances assigned for mouse MBD2_IDR_. The ^1^H chemical shifts were referenced to a separate sample of 4,4-dimethyl-4-silapentane-1-sulfonic acid (DSS) prepared in NMR buffer, while ^13^C and ^15^N chemical shifts were indirectly referenced using the IUPAC-IUB recommended ratios (40). Given the sequence identity and similar ^15^N-HSQC spectra, only HNCO, HNCACB, CBCA(CO)NH, HBHA(CO)NH and ^15^N-NOESY_HSQC triple resonance spectra were necessary to propagate backbone and sidechain H_α/β_ resonance assignments to human MBD2_IDR_. For secondary chemical shift analyses, random coil chemical shifts for the MBD2_IDR_ were calculated using the webserver at http://spin.niddk.nih.gov/bax/nmrserver/Poulsen_rc_CS/ (41).

^1^H/^15^N heteronuclear steady-state NOE spectra for human MBD2_IDR_ were collected with and without 3 s of initial proton saturation in an interleaved fashion with spectral widths of 9800 Hz over 626 complex points in ω2 (^1^H) and 1560 Hz over 256 complex points in ω1 (^15^N).

^15^N-TROSY-HSQC, TROSY-HNCO and ^15^N-NOESY-HSQC spectra were collected for a ∼1mM sample of uniform ^2^H, ^13^C, ^15^N-MBD2FLsc bound to methylated DNA. Once corrected for the spin state selective J-coupling offset, the HNCO spectra were very similar between MBD2_IDR_ and MBD2FLsc such that backbone amide resonances could be assigned for most of the IDR region of MBD2FLsc (88 out of 110 amide resonances). Based on these assignments, the weighted chemical shift distance in ppm was calculated as $\Delta = \sqrt {(0.1*\Delta \delta _N )^2 + \Delta \delta _{H_N }^2 }$.

### Cell culture

Human embryonic kidney 293T cells were maintained in Dulbecco's modified Eagle's Medium (DMEM) containing 10% heat inactivated fetal bovine serum (Hyclone), 2mM L-glutamine and 100 U/ml penicillin and streptomycin. MDA-MB-435 breast cancer cells were maintained in DMEM supplemented with 10% heat inactivated fetal bovine serum and 100 U/ml penicillin and streptomycin. Cells were cultured at 37°C and 5% CO2.

### Co-immunoprecipitation

Various MBD2 constructs were cloned into the pCMVTag2B (Stratagene) vector in frame with an N-terminal flag-tag sequence. HEK 293T cells were transfected with the resulting vectors (18 μg plasmid DNA) by calcium phosphate precipitation method (42) and harvested after 48 h. Cells were lysed and immunoprecipitated with anti-flag M2 antibody (Sigma) and mouse IgG (Santa Cruz) controls according to the Sigma Flag-IPT kit protocol (Sigma-Aldrich, Inc., St. Louis, MO, USA). The precipitated proteins were then analyzed for different components of the MBD2-NuRD complex by western blot using antibodies against RbAp48 (Abcam ab79416), HDAC2 (Millipore #05–814) and MTA2 (Santa Cruz sc-28731).

For full-length MBD2 pull downs, the cells were lysed in micrococcal nuclease (MNase) digestion buffer (25mM HEPES-KOH pH 7.6, 100mM NaCl, 5mM MgCl2, 3mM CaCl2, 10% glycerol, 0.2% NP40 and 1X EDTA-free protease inhibitor cocktail (Roche)) followed by MNase digestion using 1500 U/ml of MNase (Worthington Biochemical, Lakewood, NJ, USA) for 2 h on ice. Ethidium bromide was then added to the lysate at 300 μg/ml followed by a spin at 10 000xg for 15 mins at 4°C. The supernatant was then subjected to immunoprecipitation as previously described.

### Site-directed mutagenesis

Mutant oligonucleotides targeting the conserved residues of the 1st and 2nd region of MBD2_IDR_ were designed using the QuikChange Primer Design Program (www.agilent.com/genomics/qcpd). Primers used for mutagenesis are listed in Supplementary Table S1. Mutagenesis was carried out per manufacturer's protocol using the Quikchange Lightning Site-Directed Mutagenesis Kit (Agilent Technologies, Inc., Santa Clara, CA, USA). Clones were verified by sequencing and used for transfections.

### Sequence alignments

Protein-coding sequences of MBD2 for different species were obtained from the NCBI Protein database and aligned using the PRALINE software (http://www.ibi.vu.nl/programs/pralinewww/).

### Local isoelectric point calculation

Each residue (*i*) in the protein was assigned a local isoelectric point (pI) calculated from the amino acid sequence (43) for residues spanning *i* ± 7. The pI was calculated using the SeqUtils.IsoelectricPoint module in the Bio package from the Biopython Project (www.biopython.org). For those residues less than seven from the N- or C-termini, the isoelectric point was calculated from the amino acid sequence for residues spanning *i* ± *n* where n is the number of residues between *i* and the N- or C- terminus.

### Lentiviral knockdown and expression of MBD2

*The* shMBD2 (5′ - GGGTAAACCAGACTTGAA - 3′) sequence was cloned into the pRRL.H1.shRNA vector. Full-length wild-type and mutant MBD2 gene sequences were cloned in the pLV203 vector with a flag-tag added to the C-terminus (Genecopoeia, Rockville, MD, USA). Both full-length MBD2 genes contain a single silent mutation introduced into the MBD2 shRNA target sequence to confer shRNA resistance. The vectors were packaged into a lentivirus by calcium phosphate transfections of HEK 293T cells. MDA-MB-435 breast cancer cells were transduced with packaged virus and grown *in vitro* for 7 days post-transduction before quantitative polymerase chain reaction and western blot analyses. Protein concentrations of cell lysates were measured using the BioRad *DC*™ Protein Assay. The MBD2 protein was quantified on western blots with the *LI-COR* Odyssey® Infrared Imaging System using the Image Studio Software based on Chemi-luminescence imaging. Primers used for qPCR analysis are listed in Supplementary Table S2. Antibodies used for MBD2 and Flag epitope protein detection were from Santa Cruz (sc1244) and Abcam (ab1162), respectively. All lentiviral vector infection experiments were repeated independently at least three times and a paired Student's *t*-test used to compare repression of gene expression.

## RESULTS

### The MBD2_IDR_ region is intrinsically disordered

We cloned, expressed and purified from bacteria the MBD2_IDR_ from both the human (amino acids 238–356) and mouse (amino acids 241–359) MBD2 proteins (Figure 1a). The 2D ^15^N-HSQC spectra of each (Figure 1b) show features characteristic of a disordered peptide. The amide backbone resonances are quite sharp, the ^1^H(N) chemical shifts fall between 7.5 and 8.5 ppm, and the sidechain amide resonances are highly degenerate. In particular, the ^1^H-^15^N_ϵ_ resonances of two Trp residues completely overlap (inset, Figure 1b), while seven Asn and ten Gln sidechain ^15^N-^1^H_2_ resonances overlap extensively (blue dashed oval, Figure 1b). These findings indicate that the MBD2_IDR_ domain remains largely disordered in solution.

We further characterized this region by CD and analytical ultracentrifugation. The CD spectra are consistent with a highly disordered peptide comprised of only 9% helix at 25°C (Figure 1c) based on mean residue molar ellipticity ([θ] MRW) at 222 nm. The temperature dependence for CD at 222 nm did not show a cooperative transition typical of a folded domain (blue curve, Figure 1c). Sedimentation velocity by analytical ultracentrifugation analysis is consistent with a monomeric protein at 20 μM with a tendency to aggregate at higher concentrations (Figure 1d).

Although the backbone resonances of the MBD2_IDR_ lack significant chemical shift dispersion, we were able to assign these resonances using standard 3D heteronuclear techniques. The mouse domain was particularly well behaved, such that we first assigned mouse MBD2_IDR_ and then carried those assignments over to the human protein. Based on these assignments, secondary chemical shifts were calculated for the MBD2_IDR_ (41) and plotted for alpha (^13^C_α_) and carbonyl (^13^C’) carbons in Figure 1e. Three separate segments of the MBD2_IDR_ have positive secondary chemical shifts indicative of a propensity to form α-helices (light blue ovals, Figure 1e). For comparison, the disorder propensity for the MBD2_IDR_ as calculated by PONDR® VLXT (Predictor of Naturally Disordered Regions) (44,45) is plotted in the first panel of Figure 1e. This latter analysis predicts that two sub-regions within MBD2_IDR_ (amino acids 212–273 and 274–316) have low disorder propensity (indicated by double-headed arrows in Figure 1e). The first two of the potential helical segments fall within the second region of low disorder propensity, supporting the idea that this region can adopt transient helical secondary structure in isolation. Heteronuclear NOE values (Figure 1e, lower panel) remain below 0.5 throughout the MBD2_IDR_ indicating a high degree of internal dynamic motion; yet, the same region with low disorder propensity and large secondary chemical shifts (amino acids 212–316) also show the largest heteronuclear NOE values. Therefore, these data demonstrate that while the MBD2_IDR_ remains largely unstructured in isolation, the central portion has a tendency to form α-helices that may fold into a more stable structure either in the context of full-length protein or upon binding other components of NuRD.

To test whether the MBD2_IDR_ depends on surrounding regions of the protein to adopt a regular structure, we sought to analyze this region in the context of the surrounding MBD and coiled-coil domains. However, a polypeptide including the MBD, MBD2_IDR_ and coiled-coil domains of MBD2 has proven difficult to purify from bacterial expression systems. We recently designed and tested a remarkably stable and well-behaved single chain construct of the coiled-coil domains from MBD2 and p66α (Supplementary Figure S1a). This single-chain construct (scMBD2-p66α) comprises the MBD2 coiled-coil domain (amino acids 361–393), a short GGSG linker, and the p66α coiled-coil domain (amino acids 137–178). The scMBD2-p66α retains helical content as measured by CD analysis up to 95°C (Supplementary Figure S1b) and the 2D ^15^N-HSQC spectrum remains dispersed even at 80°C (Supplementary Figure S1c). Adding the scMBD2-p66α to the MBD and MBD2_IDR_ of MBD2 (MBD2FLsc, amino acids 212–356) stabilizes the protein for bacterial expression and purification.

**Figure 2.**
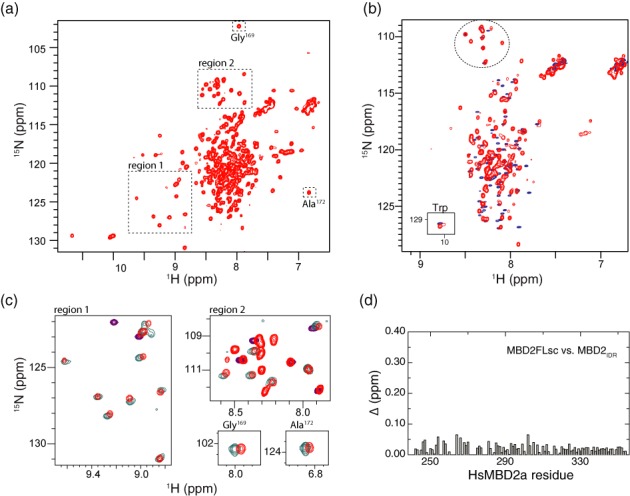
NMR analysis of intrinsically disordered MBD2_IDR_. (**a**) A 2D ^15^N-HSQC spectra of MBD2FLsc (red) bound to methylated DNA shows both sharp and highly degenerate as well as a few broadened and dispersed resonances. (**b**) An overlay of 2D ^15^N-HSQC spectra for MBD2FLsc bound to methylated DNA (red) and the MBD2_IDR_ are plotted at higher contours which shows that the sharp and degenerate resonances from the MBD2FLsc are similar to those from the MBD2_IDR_. This similarity is most easily appreciated when comparing the more isolated peaks from glycine residues (highlighted with a dashed circle). (**c**) Expanded regions from the 2D ^15^N-HSQC show that the broadened and dispersed resonances from MBD2FLsc (red) align with resonances from either the MBD2_MBD_ bound to methylated DNA (teal) or the MBD2-p66α coiled-coil complex (purple). Likewise, two key resonances from the MBD2_MBD_ that show unique chemical shifts when bound to methylated DNA (Gly169 and ALa172) show similar chemical shifts in the context of MBD2FLsc. (**d**) Plotting the chemical shift distance (Δ) between resonances from the isolated MBD2_IDR_ and the same region of MBD2FLsc when bound to methylated DNA shows only small chemical shift differences (< 0.05 ppm) throughout the region.

A 2D ^15^N-HSQC spectrum of the MBD2FLsc protein bound to methylated DNA contains a few broadened and dispersed as well as many sharp and highly degenerate resonances (Figure 2a). An overlay of the 2D ^15^N-HSQC spectra (Figure 2b) for MBD2FLsc bound to methylated DNA (red) and MBD2_IDR_ (blue), plotted at a higher contour level, shows that the sharp and degenerate resonances from MBD2FLsc are similar to those of the MBD2_IDR_. Given the similarity in chemical shifts and the sharpness of these peaks, we were able to carry over assignments for 80% of the amide resonances of the IDR in the MBD2FLsc:DNA complex. Plotting ^1^H,^15^N chemical shift differences between resonances from the isolated MBD2_IDR_ and the same region in the MBD2FLsc:DNA (Figure 2d) confirm that the chemical shifts are very similar throughout the region (Δ < 0.05ppm).

Comparing the broadened and more dispersed resonances (Figure 2c) with those for the isolated MBD2_MBD_ bound to methylated DNA (teal) and coiled-coil domains (purple) (18) shows that these peaks are attributable to the MBD and coiled-coil domains. In previous studies we found that the chemical shifts for Gly^169^ and Ala^172^ are quite different when bound to either unmethylated or methylated DNA: Gly^169^ shifts from 107 ppm to 102 ppm in ^15^N while the Ala^172^ shifts from 7.0 ppm to 7.3 ppm in ^1^H_N_, respectively (10). As can be seen in Figure 2c, Gly^169^ and Ala^172^ show very similar chemical shifts for both MBD2FLsc and MBD2_MBD_ indicating a similar methylation specific binding mode. Together these findings show that the MBD2_IDR_ region remains largely disordered even in the context of full-length protein and does not appear to affect the structure or DNA binding mode of the MBD.

### The MBD2_IDR_ modifies DNA binding kinetics and overall binding affinity

**Figure 3.**
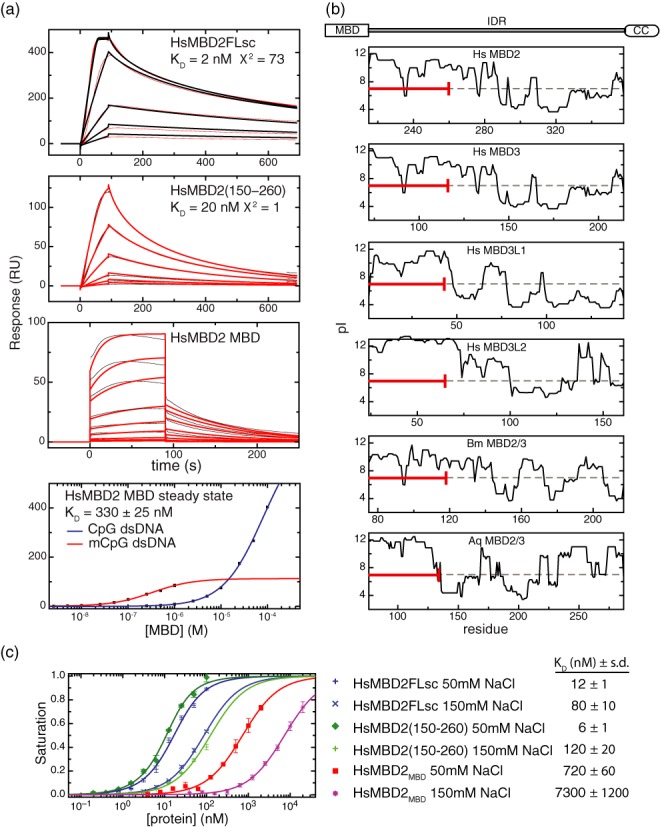
The MBD2_IDR_ modifies DNA binding kinetics and affinity. (**a**) Surface plasmon analysis of DNA binding shows that both MBD2FLsc (upper panel), and MBD2(150–260) (second panel) bind with higher affinity than the isolated MBD (third panel). Steady-state analysis of the isolated MBD binding to methylated (red) and unmethylated (blue) DNA is shown in the fourth panel. (**b**) The local isoelectric point, calculated as described in the text, is plotted for the region homologous to the MBD2_IDR_ in *Hs*MBD2, *Hs*MBD3, *Hs*MBD3-L1, *Hs*MBD3-L2, *Bm*MBD2/3 and *Aq*MBD2/3. A red bar indicates the region from the N-terminus of the MBD2_IDR_ included in the MBD2(150–260) construct, which shows that the positive charge is conserved across the animal kingdom. (**c**) To validate the SPR studies, binding affinity (*K_D_*) of MBD2FLsc, MBD2(150–260) and MBD2_MBD_ for methylated DNA was determined by a fluorescence polarization assay. Fitting the resulting binding curves to a general two-state binding model shows that both MBD2FLsc and MBD2(150–260) bind with ∼100-fold greater affinity than the isolated MBD2_MBD_. Furthermore, the binding affinity of each shows a similar dependence on salt concentration such that increasing NaCl from 50 to 150 mM reduces the affinity by ∼10-fold.

To determine if the MBD2_IDR_ alters the affinity of MBD2 for DNA, we measured binding to methylated DNA by surface plasmon resonance. As described previously (33), the isolated MBD shows rapid on and off-rates, which requires steady state analysis to determine the overall binding affinity (*K_D_* = 330 nM, Figure 3a). In contrast, the MBD2FLsc shows an ∼100-fold increase in affinity (*K_D_* = 2 nM) as compared to the MBD2_MBD_. Inspection of the MBD2_IDR_ reveals that the region just C-terminal to the MBD contains positively charged Arg and Lys residues, which could contribute to DNA binding through non-specific electrostatic interactions. Therefore, we calculated a theoretical isoelectric point for a sliding window of 15 amino acids along the entire length of each protein. The results of this analysis for human (*Hs*) MBD2 paralogs (MBD2, MBD3, MBD3-L1 and MBD3-L2) as well as *Bombyx mori (Bm*) and *Amphimedon queenslandica (Aq*) MBD2 orthologs are plotted for the region homologous to the MBD2_IDR_ of each protein in Figure 3b. Each of the MBD2 orthologs contains a positively charged region just following the MBD and at the N-terminus of the MBD2_IDR_ (Figure 3b). To test whether this region is sufficient to modify DNA binding, we measured binding kinetics by surface plasmon resonance for an MBD2 construct that includes the MBD and the first 20 amino acids of the MBD2_IDR_ (residues 150–260). This construct incorporates amino acids from the N-terminal portion of the IDR (red bar, Figure 3b) that show evolutionary conservation of a net positive charge. Similar to MBD2FLsc, MBD2(150–260) binds with increased affinity (*K_D_* = 20 nM) compared to the isolated MBD (Figure 3a).

To validate these findings, we measured DNA binding affinity using a fluorescence polarization based assay (Figure 3c). Similar to the results from SPR studies, MBD2FLsc and MBD (150–260) bind methylated DNA with ∼100-fold greater affinity than MBD2_MBD_. In addition, the binding affinity for all three constructs decreases by ∼10-fold with an increase in NaCl concentration from 50 mM to 150 mM. The latter observation is consistent with non-specific charge interactions contributing to the overall binding affinity. Therefore, the MBD2_IDR_ contains a positively charged region that modifies binding kinetics and affinity despite a lack of regular structure formation. Similar positively charged disordered regions adjacent to DNA binding domains have been described previously and referred to as ‘fuzzy’ domains (46–48).

### The MBD2_IDR_ binds the histone deacetylase core complex of NuRD

**Figure 4.**
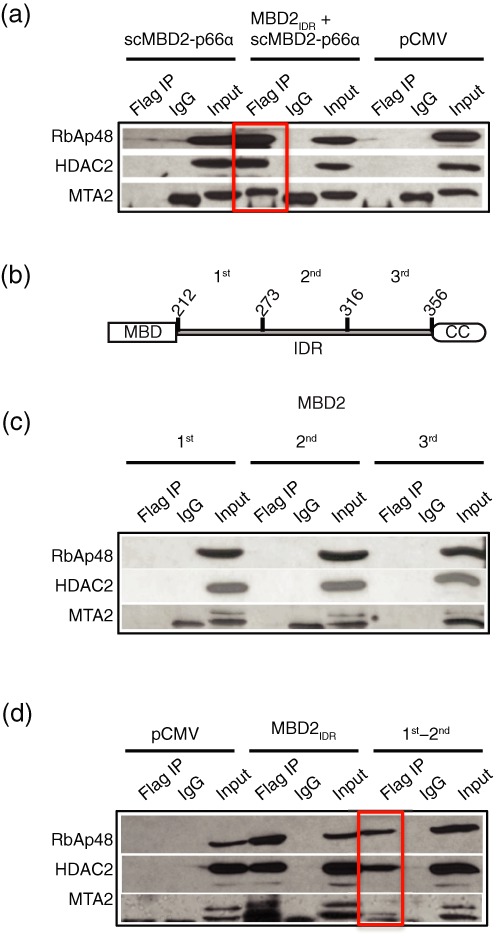
The MBD2_IDR_ binds the histone deacetylase core complex of NuRD. (**a**) The MBD2_IDR_ with scMBD2-p66α was transfected in high-transfection-efficiency HEK 293T cells. Immunoprecipitation and western blot analysis of the transfected cells indicates that the MBD2_IDR_ binds the histone deacetylase core components RbAp48, HDAC2 and MTA2, whereas the scMBD2-p66α construct does not. The flag-IP lane shows pull down of the histone deacetylase core components by immunoprecipitation using an anti-flag antibody directed against the flag-tagged MBD2_IDR_. IgG and expression vector pCMV serve as negative controls while the input lane shows 2% of the input. (**b**) The MBD2_IDR_ was divided into three sub-regions to test whether we could isolate critical binding region(s) for the core complex components. (**c**) The three sub-regions of MBD2_IDR_ were expressed individually in HEK 293T cells and immunoprecipitated, but failed to bind to either of the histone deacetylase core complex components. Note that a non-specific band appears in the IgG lane when blotted with anti-MTA2 that runs just below the MTA2 protein in the input and flag IP lanes. (**d**) The region of MBD2_IDR_ from amino acids 212–316 comprising the first and second ordered sub-regions in combination can bind to RbAp48, HDAC2 and MTA2, although a weaker interaction with MTA2 was observed.

In previous studies we showed that the coiled-coil domain of MBD2 recruits the p66α and CHD4 proteins to NuRD. To investigate whether the MBD2_IDR_ and coiled-coil domains interact with the remaining core components of NuRD (MTA2, HDAC2 and RbAp48), the MBD2_IDR_ was expressed in 293T cells as a flag-tagged construct, with and without the scMBD2-p66α coiled-coil domains (Figure 4a and d, respectively). Cell lysates were immunoprecipitated with an anti-flag antibody followed by western blot analysis to identify the NuRD components interacting with the flag-tagged MBD2_IDR_. Figure 4a shows that the MBD2_IDR_ of MBD2 strongly interacts, either directly or indirectly, with the RbAp48, HDAC2 and MTA2 components of NuRD while the scMBD2-p66α does not. The MBD2_IDR_ does not interact with p66α and CHD4 proteins (Supplementary Figure S2) as expected, which uniquely maps the interaction of MBD2 with the histone deacetylase and chromatin remodeling components of the NuRD complex into two distinct functional domains, the MBD2_IDR_ and the MBD2 coiled-coil domain, respectively.

Segments of intrinsically disordered proteins can fold upon interacting with target proteins to adopt regular secondary structure. These segments, referred to as molecular recognition features (MoRFs)(48–50), may be identified as regions with low disorder propensity by disorder prediction algorithms. To identify the minimal region necessary for binding to the histone deacetylase core complex of NuRD, the MBD2_IDR_ was divided into three separate sub-regions (amino acids 212–273, 274–316 and 317–360, Figure 1e) in order to isolate the central portion with helical and structural propensity from the N and C-terminal regions. We tested whether each of these regions, separately and in combination, could immunoprecipitate HDCC components. As can be seen in Figure 4c, none of these three sub-regions interact with the core complex in isolation. However, the first and second sub-regions of MBD2_IDR_ in combination (amino acids 212–316) were sufficient to bind RbAp48, HDAC2 and MTA2, albeit with a somewhat weaker interaction with MTA2 as compared to the entire MBD2_IDR_ (Figure 4d).

Key residues within minimal MBD2_IDR_ are evolutionarily conserved: full-length protein sequences of HsMBD2 orthologs from across the animal kingdom (Aq, Trichoplax adhaerans (Ta), Bm, Danio rerio (Dr), Gallus gallus (Gg), Mus musculus (Mm)), and Hs paralogs MBD3, MBD3-like 1 and 2 (MBD3-L1, MBD3-L2) were obtained from the NCBI protein database. The protein sequences of individual domains of MBD2 were aligned using the default settings of the PRALINE online server. An alignment of the MBD (Supplementary Figure S3a) for orthologs (Aq, Bm, Dr, Gg and Mm) and a paralog (MBD3) that contain a recognizable MBD shows a high degree of conservation from sponge to human (AqMBD2 and HsMBD2 proteins share 64% identity in their MBDs). Critical residues known to influence DNA binding affinity and methylation selectivity (Tyr^178^ and Lys^174^ of HsMBD2) (10,12,13) are highly conserved indicating that methylation specificity likely developed with the first multi-cellular organisms. Notably, several homologs (Ta MBD2/3, Hs MBD3-L1 and L2) lack an MBD, yet previous studies have shown that both the MBD3-L1 and L2 paralogs interact with NuRD and compete with MBD2 and MBD3 for complex formation (51–53). Therefore, NuRD complex recruitment and DNA binding represent distinct and separable functions of MBD2.

**Figure 5.**
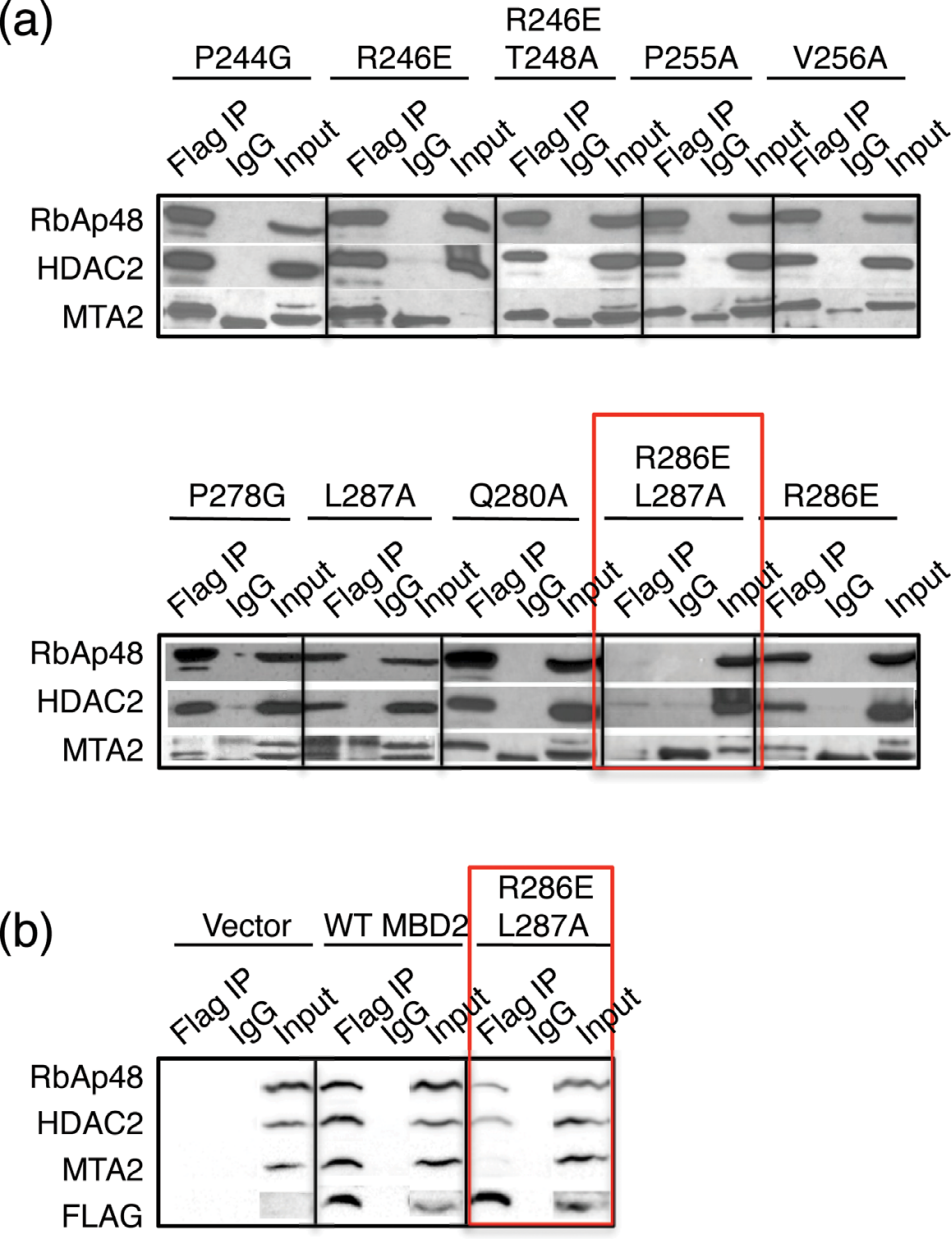
Two conserved residues from minimal MBD2_IDR_ are necessary to bind the histone deacetylase core of NuRD. (**a**) Mutating five residues from the first region of minimal MBD2_IDR_ (P244G, R246E, P255A, V256A and R246E/T248A) does not affect immunoprecipitation of the histone deacetylase core complex. Similarly, mutating four residues from the second ordered region of minimal MBD2_IDR_ (P278G, Q280A, R286E and L287A) does not affect immunoprecipitation of the histone deacetylase core complex. However, combined mutation of two adjacent residues (R286E/L287A) significantly abrogates the ability of MBD2_IDR_ to recruit the histone deacetylase core components. Strikingly, the individual mutants R286E and L287A have no effect on interaction of the MBD2_IDR_ with the histone deacetylase core. Three additional mutations (T248A, W283A and L290A) do not affect binding of MBD2_IDR_ to the histone deacetylase core complex (data not shown). (**b**) Full length flag-tagged MBD2 carrying the double mutation R286E/L287A (Double Mutant) also displays a disrupted interaction with RbAp48, HDAC2 and MTA2. The interaction of Double Mutant MBD2 with MTA2 is completely lost whereas its interaction with RbAp/HDAC shows significant reduction.

An alignment of the MBD2_IDR_ and coiled-coil regions (Supplementary Figure S3b), shows much less conservation with large insertions (i.e. *Ta*MBD2/3 and *Aq*MBD2/3) and smaller deletions as compared to *Hs*MBD2. Nonetheless, specific individual residues are absolutely conserved across species suggesting that these amino acids are critical to function. Highly conserved residues were identified within the first two ordered regions of MBD2_IDR_ implicated in binding to the NuRD histone deacetylase core complex. We mutated these conserved residues individually or in pairs as follows: (1) P244G, (2) R246E, (3) T248A, (4) P255A, (5) V256A, (6) P278G, (7) Q280A, (8) W283A, (9) R286E, (10) L287A, (11) L290A, (12) R246E/T248A and (13) R286E/L287A (see red stars above the sequence alignment in Supplementary Figure S3b). Flag-tagged MBD2_IDR_ mutants were expressed in 293T cells followed by immunoprecipitation and blotting for components of the histone deacetylase core complex of NuRD. Of the eleven conserved residues tested, only two contiguous amino acids from the second ordered region of MBD2_IDR_, Arg^286^ and Leu^287^, when mutated, markedly decreased binding to the core complex (Figure 5a). Interestingly neither of these mutations in isolation nor any of the other mutations disrupted binding to the histone deacetylase core components. Next, we introduced the double mutations, R286E/L287A, into the full-length human MBD2 gene sequence by site-directed mutagenesis. Since MBD2-NuRD contains several potential DNA binding proteins, the immunoprecipitation protocol for full length MBD2Flag constructs was modified to eliminate contamination through non-specific DNA binding. The cell lysate from transfected cells was treated with micrococcal nuclease, which cleaves both single- and double-stranded DNA and RNA, and with ethidium bromide, which intercalates between stacked bases in the double helix. As expected, immunoprecipitation of the NuRD complex by the mutant full length MBD2 revealed impaired interaction with the histone deacetylase core components: RbAp48, HDAC2 and MTA2, with the effect being most pronounced for MTA2 (Figure 5b). Together these observations indicate that Arg^286^ and Leu^287^ comprise a critical interaction surface necessary but not sufficient for recruiting the histone deacetylase core complex of NuRD.

To determine if interaction with the histone deacetylase core components is necessary for methylation dependent gene silencing by MBD2, we designed a repression assay to study the functional effects of the R286E/L287A (Double Mutant) MBD2 on expression of an endogenous gene. The Double Mutant MBD2Flag construct was expressed in a background of partial knockdown of endogenous MBD2 in MDA-MB-435 breast cancer cells. The percentage of repression of the endogenous tumor suppressor gene *PRSS8* was used to measure MBD2-NuRD mediated transcriptional repression. We previously showed that 75% stable knockdown of MBD2 by shRNA in MDA-MB-435 cells increased *PRSS8* expression, demonstrating that MBD2 mediates endogenous transcriptional repression of this gene in these cells (20).

**Figure 6.**
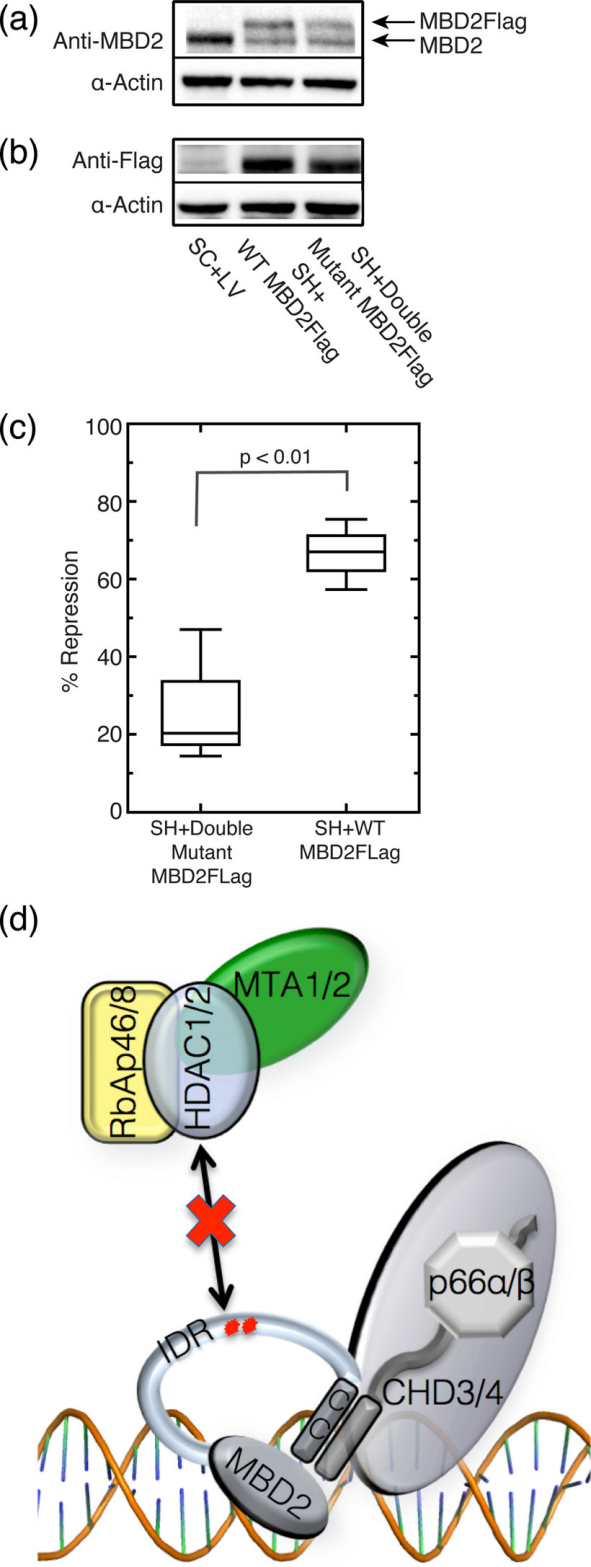
Double Mutant MBD2 shows reduced transcriptional repression of its methylated target gene *PRSS8*. Western blot quantitative chemiluminescence analysis of MDA-MB-435 cells infected with lentivirus for MBD2 knockdown (SH) and expression of wild-type (WT) and R286E/L287A (Double Mutant) MBD2 shows (**a**) ∼50% and ∼40% knockdown of endogenous full-length MBD2 in SH+WT and SH+Double Mutant cells, respectively, and (**b**) equivalent expression of WT and Double Mutant MBD2Flag in SH+WT and SH+Double Mutant cells, respectively. SH+Double Mutant cells contain ∼60% mutant MBD2Flag as compared to SH+WT cells. (**c**) Transcript levels of *PRSS8* were measured by qPCR, normalized to endogenous transcript levels of *GAPDH*, and plotted as percent repression compared to SC+LV control cells. SH+WT cells show significantly increased repression of *PRSS8* as compared to SH+Double Mutant cells (p < 0.01). Box plots represent three independent experiments and a paired Student's *t*-test used to compare repression of *PRSS8* gene expression. (**d**) A working model of the architecture of the MBD2-NuRD co-repressor complex maps interactions between MBD2 and the histone deacetylase core subunits to the MBD2_IDR_, wherein mutation of the two contiguous residues comprising the critical interaction site (shown as red asterisks in the IDR) impairs binding. The previously characterized (CC) coiled-coil domain of MBD2 recruits the chromatin remodeler CHD4 through p66α and the MBD recruits the co-repressor complex to sites of dense CpG methylation.

Concomitant knockdown of endogenous MBD2 and enforced expression of wild-type or Double Mutant MBD2Flag was achieved by simultaneous lentivirus mediated delivery of anti-MBD2 shRNA (SH) and wild-type (WT) or Double Mutant MBD2Flag mRNA resistant to knockdown (20). Western blotting with anti-MBD2 antibody (Figure 6a), which recognizes both endogenous (lower band) and lentivirally expressed MBD2Flag (top band), shows about 50% knockdown of the endogenous MBD2 protein in SH+WT and SH+Double Mutant cells as compared to negative control cells simultaneously infected with both a scramble short RNA sequence (SC) expression vector and an empty lentivirus (LV) expression vector. Despite the ability of this MBD2 shRNA construct to knock down MBD2 levels by greater than 75% (Supplementary Figure S4a) (20), we were unable to achieve the same level of knockdown when cells were concomitantly infected with lentiviral MBD2 expression constructs, regardless of whether cells were infected with both constructs simultaneously or sequentially (in either order). Detection of the exogenously expressed MBD2Flag proteins with an anti-flag antibody demonstrates similar expression levels in SH+WT and SH+Double Mutant transduced cells, as determined by quantitative chemiluminescence (Figure 6b). Taking into account both knockdown of endogenous MBD2 and expression of MBD2Flag proteins, the total amount of MBD2 protein in both groups (SH+WT and SH+Double Mutant) remains constant and represents more total MBD2 protein than in the negative control cells. Partial knockdown of endogenous MBD2 and enforced expression of wild type MBD2, results in approximately 70% more repression of the *PRSS8* gene than in negative control cells. However, enforced expression of the Double Mutant MBD2 results in a smaller increase in repression of the *PRSS8* gene as compared to the negative control cells (Figure 6c). Hence the amount of repression of the *PRSS8* gene is consistently ≥2-fold higher (*P*-value<0.01) in SH+WT cells than in SH+Double Mutant cells. We attribute this difference to the inability of Double Mutant MBD2 to effectively recruit the histone deacetylase core components of the NuRD complex. The suggestion of residual repressive activity by the Double Mutant MBD2 may reflect that it retains an intact coiled-coil domain. We have shown previously that the interaction of the coiled-coil domain with p66α and CHD4 mediates at least part of the repressive activity of MBD2-NuRD (18).

## DISCUSSION

Research over the past two decades has challenged the traditional structure-function paradigm of proteins. Instead of adopting a well-folded three-dimensional structure in solution, many proteins are entirely disordered or contain functionally important intrinsically disordered regions (IDR). Estimates indicate that from 15% to 45% of eukaryotic proteins have long (>30 amino acid) regions of disorder (28). These regions can function either by folding upon binding to a target ligand or through dynamic interactions involving rapidly exchanging conformations of the disordered state (28,46,48).

Here we show that a large segment (∼145 amino acids) between the conserved MBD and coiled-coil domains of MBD2 behaves as an intrinsically disordered region in isolation and in the context of the full-length protein. This region does not adopt a regular structure even when bound to methylated DNA. Nonetheless incorporating the MBD2_IDR_ affects the binding kinetics and overall affinity for DNA, functioning as a ‘fuzzy’ domain (46). The N-terminal portion of the MBD2_IDR_ has a net positive charge that is conserved across evolution and likely contributes to this fuzzy interaction with the DNA (see Figure 3b). IDRs flanking functional DNA-binding domains have been shown to influence DNA recognition and potentially rates of association and lifetime of the DNA-protein interactions (54,55).

IDRs are often found in proteins that serve as hubs of one-to-many protein interactions (56–58). In this report we demonstrate that the MBD2_IDR_ of MBD2 binds the histone deacetylase core complex of NuRD comprised of the MTA2, HDAC2 and RbAp48 proteins. To map this interaction, we divided the MBD2_IDR_ into three potential MoRFs based on NMR analyses of residual structure and the disorder propensity. We found that the first two segments of the MBD2_IDR_ are necessary and sufficient to bind the histone deacetylase core complex (Figure 4d). Interestingly, this region incorporates both the positively charged segment that contributes to DNA binding as well as the region with helical propensity. We were unable to identify sub-regions of MBD2_IDR_ that selectively bind to different components of the histone deacetylase core complex, which suggests cooperative all-or-none formation of this complex (Figure 4c). This observation indicates that either the MBD2_IDR_ forms a large contact surface for binding each of the proteins cooperatively or that the MBD2_IDR_ binds to one of the core components which in turn interacts with other components. In support of the latter interpretation, recent structural analyses have shown that the ELM2 and SANT domains of MTA1 bind directly to HDAC1 (59) and a small peptide from the C-terminal region of MTA1 binds RbAp48 (60). The MTA proteins may provide a common point of contact such that MBD2_IDR_ binding directly to either of RbAp, HDAC, or MTA would be sufficient to recruit all of the histone deacetylase core components.

Intrinsic disorder in MBD2 appears to be under selective pressure as evidenced by conservation of numerous residues within the MBD2_IDR_ across several paralogs and distantly related orthologs, despite large insertions and sequence variability (Supplementary Figure S3). Using this evolutionary conservation as a guide, we designed point mutations in the first and second segments of the MBD2_IDR_ and found that two highly conserved residues (Arg^286^ and Leu^287^) located within the helical region of the MBD2_IDR_ are critical for binding to the histone deacetylase core complex. Mutating these residues even in the context of full length MBD2 diminishes interaction with the histone deacetylase core complex. A recent analysis of MBD2 alternative splice variants revealed that dominant isoform in embryonic stem cells, MBD2c, lacks most of the C-terminus including the MBD2_IDR_ and the coiled-coil domains. Consistent with the role of these two domains in recruiting CHD4 and the histone deacetylase core complex, MBD2c fails to interact with any of the NuRD components and lacks repressive activity (61).

MBD2-NuRD has been established as a transcriptional repressor of embryonic and fetal β-type globin genes in primary adult erythroid cells across different species (4,5,17,18) and of tumor suppressor genes in breast cancer cells (20,26). We hypothesized that the Arg^286^ and Leu^287^ mutations of MBD2 that impair recruitment of the histone deacetylase core components of the NuRD complex would in turn abrogate repression of methylated target genes. To test this hypothesis, we assayed the expression of a previously identified highly methylated MBD2 target gene, *PRSS8*, in MDA-MB-435 breast cancer cells (20). *PRSS8* encodes for prostasin, a member of the trypsin family of serine proteases that has been implicated in inhibition of metastasis of both breast and prostate cancer cells (62,63). A CpG-rich region in the promoter and exon 1 of *PRSS8* is heavily methylated when this gene is repressed in MDA-MB-435 breast cancer cells (62), and demethylation by 5-aza-2′-deoxycytidine combined with histone deacetylase inhibitor treatment reactivates expression of *PRSS8*. In our studies, Double Mutant MBD2 protein exerts diminished repression of *PRSS8* when compared to an equivalent amount of wild-type MBD2 protein, (Figure 6c) thus confirming the importance of these two amino acid residues in MBD2-NuRD function.

Our results can be summarized into a dynamic working model for the architecture of the NuRD complex wherein the subunits carrying the chromatin remodeling enzymatic activities unique to the NuRD complex can be mapped to two separate domains of MBD2: the MBD2_IDR_ which recruits the histone deacetylase core components and the coiled-coil domain which recruits the chromatin remodeling subunit CHD4 through its interaction with p66α (Figure 6d). Delineating the multi-protein interactions within the MBD2-NuRD complex should enable future attempts to decouple the distinct enzymatic activities of the complex, in isolation or combination, to disrupt the repressive functions of each and dissect their potentially independent roles in regulating specific sets of genes.

Understanding the role of IDRs in the assembly of multi-protein complexes like MBD2-NuRD adds valuable insight to their function in epigenetic regulation. IDRs have been well recognized as hubs for interaction of many proteins due to their malleability and fluctuating structures. MBD1-c was recently shown to selectively interact with different binding partners through its intrinsically disordered transcriptional repression domain (29). Structural characterization of intrinsically disordered regions of other proteins such as p53 (64,65), PTP1B (66), androgen receptor (67), α-synuclein (68,69) and oncoprotein c-myc (70,71) has elucidated the function of these proteins in pathological pathways and lead to successful efforts developing small molecule inhibitors for therapeutic drug targeting. The work presented here sets the stage for further biochemical and functional characterization of the MBD2-NuRD complex and raises the possibility of selectively abrogating NuRD dependent silencing of specific sets of genes by disrupting the MBD2_IDR_ mediated interactions with the HDAC complex. Exploitation of such mechanistic findings could ultimately lead to improved treatment of hemoglobin disorders and cancer.

## ACCESSION NUMBERS

The NMR assignments have been deposited in the Biological Magnetic Resonance Bank (BMRB accession: 25426).

## SUPPLEMENTARY DATA

Supplementary Data are available at NAR Online.

SUPPLEMENTARY DATA
