# Supplementary material for: An intrinsically disordered region of methyl-CpG binding domain protein 2 (MBD2) recruits the histone deacetylase core of the NuRD complex
Source: Nucleic Acids Res. 2015 Mar 9;43(6):3100–13. doi: 10.1093/nar/gkv168 (PMC4381075; doi:10.1093/nar/gkv168)
Supplement: SUPPLEMENTARY DATA [file supp_43_6_3100__index.html]

An intrinsically disordered region of methyl-CpG binding domain protein 2 (MBD2) recruits the histone deacetylase core of the NuRD complex — An intrinsically disordered region of methyl-CpG binding domain protein 2 (MBD2) recruits the histone deacetylase core of the NuRD complex — SUPPLEMENTARY DATA 

# An intrinsically disordered region of methyl-CpG binding domain protein 2 (MBD2) recruits the histone deacetylase core of the NuRD complex

## SUPPLEMENTARY DATA

**Files in this Data Supplement:**

- SUPPLEMENTARY DATA
