# Supplementary material for: An intrinsically disordered region of methyl-CpG binding domain protein 2 (MBD2) recruits the histone deacetylase core of the NuRD complex
Source: Nucleic Acids Res. 2015 Mar 9;43(6):3100–13. doi: 10.1093/nar/gkv168 (PMC4381075; doi:10.1093/nar/gkv168)
Supplement: SUPPLEMENTARY DATA [file supp_gkv168_nar-02898-m-2014-File008.pdf]

Table S1. List of primers used for site-directed mutagenesis.

	FORWARD	REVERSE
P244G	GAAAATTGATGCTGTTTGTCTAATTCC CAATGTTGTATTCAAGTCTGGTTTA	TAAACCAGACTTGAATACAACATTGG GAATTAGACAAACAGCATCAATTTTC
R246E	CCAGACTTGAATACAACATTGCCAAT TGAGCAAACAGCATCAATTTTCAAAC AACC	GGTTGTTTGAAAATTGATGCTGTTTG CTCAATTGGCAATGTTGTATTCAAGT CTGG
T248A	GACTTGAATACAACATTGCCAATTAG ACAAGCAGCATCAATTTTCAA	TTGAAAATTGATGCTGCTTGTCTAATT GGCAATGTTGTATTCAAGTC
R246E/T2 48A	GGTTGTTTGAAAATTGATGCTGCTTG CTCAATTGGCAATGTTGTATTCAAGT CTGGTTTACC	GGTAAACCAGACTTGAATACAACATT GCCAATTGAGCAAGCAGCATCAATTT TCAAACAACC
P255A	GACAAACAGCATCAATTTTCAAACAA GGGGTAACCAAAGTCACAAATCAT	ATGATTTGTGACTTTGGTTACCCCTT GTTTGAAAATTGATGCTGTTTGTC
V256A	CATCAATTTTCAAACAACCGGCAACC AAAGTCACAAATCATCC	GGATGATTTGTGACTTTGGTTGCCGG TTGTTTGAAAATTGATG
P278G	ACAACGAATGAATGAACAGGGACGTC	CTCCCAGAAAAGCTGACGTCCCTGTT
Q280A	CGAATGAATGAACAGCCACGTGCGC T	CTTCTCCCAGAAAAGCGCACGTGGCT
W283A	AGCCACGTCAGCTTTTCGCGGAGAA G	CTTGTAGCCTCTTCTCCGCGAAAAGC
R286E	TCAGCTTTTCTGGGAGAAGGAGCTAC	TGCACTAAGTCCTTGTAGCTCCTTCT
L287A	GCTTTTCTGGGAGAAGAGGGCACAA	TGATGCACTAAGTCCTTGTGCCCTCT
R286E/L2 87A	CACGTCAGCTTTTCTGGGAGAAGGA G	CATCTGATGCACTAAGTCCTTGTGCC
L290A	GGAGAAGAGGCTACAAGGAGCTAGT	TGTTACATCTGATGCACTAGCTCCTT
**For full-length MBD2 mutagenesis of specific residues, following primers were used:**		
R286E/L2 87A	TTT TCT GGG AGA AGG AGG CAC AAG	CTT TTC TGG GAG AAG GAG CTA CAA G
R286E	CTT TTC TGG GAG AAG GAG CTA CAA G	CTT TTC TGG GAG AAG GAG CTA CAA G

Table S2. List of primers used for qPCR.

	FORWARD	REVERSE	PROBE
MBD2	AAG AGC GAG TAC AGC AAG TAC GCA	TTC TGT ATC AGC AGC TCG CGA CAA	TGG AAG AAG CAC TGA TGG CAG ACA TC
GAPDH	TCG ACA GTC AGC CGC ATC TTC TTT	ACC AAA TCC GTT GAC TCC GAC CTT	AGC CAC ATC GCT CAG ACA CCA TGC
PRSS8	ACGCCTTCATAGG TGATGCT	ATCTTGGATTACTC CGGTCG	

FIGURE S1. A polypeptide linkage stabilizes the MBD2-p66α coiled-coil complex. (a) A ribbon diagram depicts a model of the scMBD2-p66α derived from the previously determine solution structure of the MBD2-p66α coiled-coil complex (PDB ID: 2I2I) (1). The single chain construct consists of the MBD2 coiled-coil domain (cyan), a short GGSG linker (yellow), and the p66α coiled-coil domain (blue). (b) CD spectra were collected from 190-260 nm (0.5 nm interval, 50 nm/min, 1 cm path length, 20 ºC) on a JASCO J-715 CD spectrometer (JASCO Corp). This analysis shows that the scMBD2-p66α contains similar helical content at 25 ºC and 95 ºC. The temperature dependence of CD was followed by measuring ellipticity at 222 nm at 1 ºC intervals over a temperature range of 5-95 ºC with a heating rate of 1 ºC /min. This thermal melt shows that the coiled-coil complex does not undergo a cooperative unfolding transition. (c) An overlay of ^{15}N-HSQC spectra for the scMBD2-p66α (gray) with the MBD2 (red) and p66α (blue) coiled-coil complex peptides shows similar dispersion and chemical shifts. (d) ^{15}N-HSQC spectra of scMBD2-p66α were collected at 25 (gray), 50 (blue), and 85 (orange) ºC and referenced to H_2O (adjusted for temperature). An overlay shows that the spectra remain well dispersed indicating that the scMBD2-p66α resists thermal denaturation.

Figure S2. $MBD2_{IDR}$ does not interact with p66α and Mi2β subunits of NuRD. Immunoprecipitation of $MBD2_{IDR}$ and the $MBD2_{IDR}$ minimal binding region in transfected

HEK293T cells shows that the $MBD2_{IDR}$ does not interact with p66α and Mi2β subunits of the NuRD complex. These results delineate unique points of contact associating $MBD2_{IDR}$ with the histone deacetylase core subunits and, in conjunction with our previous data, the coiled-coil domain with the chromatin remodeling component(s) of the NuRD complex.

FIGURE S3. Evolutionary conservation of key residues within minimal $MBD2_{IDR}$. (a) The protein sequence of the MBD of MBD2 for orthologs (*Aq, Bm, Dr, Gg and Mm*) and a paralog (MBD3) were aligned with the PRALINE program online (http://www.ibi.vu.nl/programs/pralinewww/). The software color codes the most conserved residues on a scale of 0 to 10 with 10 being the most conserved residue. Most residues within the MBD show high degree of conservation including critical residues known to influence DNA binding affinity and methylation selectivity. (b) Alignment of the protein sequence of $MBD2_{IDR}$ and the coiled-coil domain shows much less conservation with large insertions (*Ta*MBD2/3 and *Aq*MBD2) and smaller deletions as compared to *Hs*MBD2. However, absolute conservation of specific residues in the minimal $MBD2_{IDR}$ suggests functional importance. The conserved residues identified for functional analysis (with a conserved score of 8 out of 10) are indicated by a red star above the sequence alignment and include: Pro^{244}, Arg^{246}, Thr^{248}, Pro^{255}, Val^{256}, Pro^{278}, Gln^{280}, Trp^{283}, Arg^{286}, Leu^{287}, and Leu^{290}.

Figure S4: (a) Knockdown of MBD2 protein in MDA-MB-435 breast cancer cells. Lentiviral infection of the breast cancer cells with anti-MBD2 shRNA results in about 70% knockdown of MBD2 protein using β–actin as the loading control. (b) Western blot shows a slight increase in expression of the Double Mutant as compared to WT $MBD2_{IDR}$ with β–actin expression as a loading control.

1. Gnanapragasam,M.N., Scarsdale,J.N., Amaya,M.L., Webb,H.D., Desai,M.A., Walavalkar,N.M., Wang,S.Z., Zu Zhu,S., Ginder,G.D. and Williams,D.C. (2011) p66Alpha-MBD2 coiled-coil interaction and recruitment of Mi-2 are critical for globin gene silencing by the MBD2-NuRD complex. *Proc. Natl. Acad. Sci. U. S. A.*, **108**, 7487–7492.

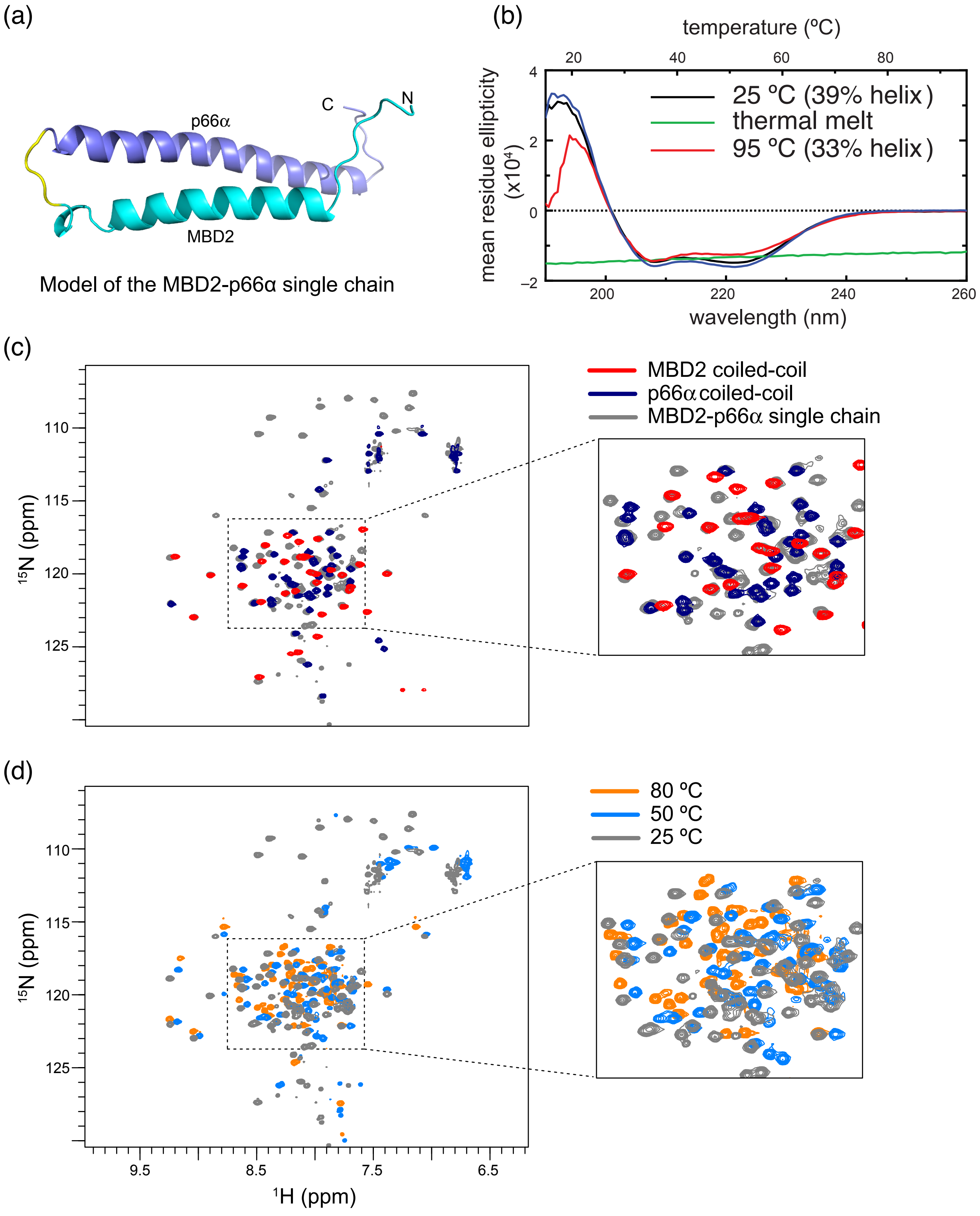
(a)
p66α
C
N
MBD2
Model of the MBD2-p66α single chain
(b)
temperature (ºC)
20
40
60
80
mean residue ellipticity (x10⁴)
4
2
0
−2
25 ºC (39% helix)
thermal melt
95 ºC (33% helix)
200
220
240
260
wavelength (nm)
(c)
MBD2 coiled-coil
p66α coiled-coil
MBD2-p66α single chain
¹⁵N (ppm)
110
115
120
125
(d)
80 ºC
50 ºC
25 ºC
¹⁵N (ppm)
110
115
120
125
9.5
8.5
7.5
6.5
¹H (ppm)

Figure S1

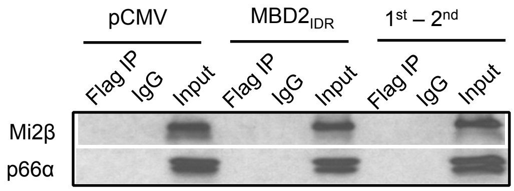
pCMV
MBD2$_{IDR}$
1st – 2nd
Flag IP
IgG
Input
Flag IP
IgG
Input
Flag IP
IgG
Input
Mi2β
p66α

Figure S2

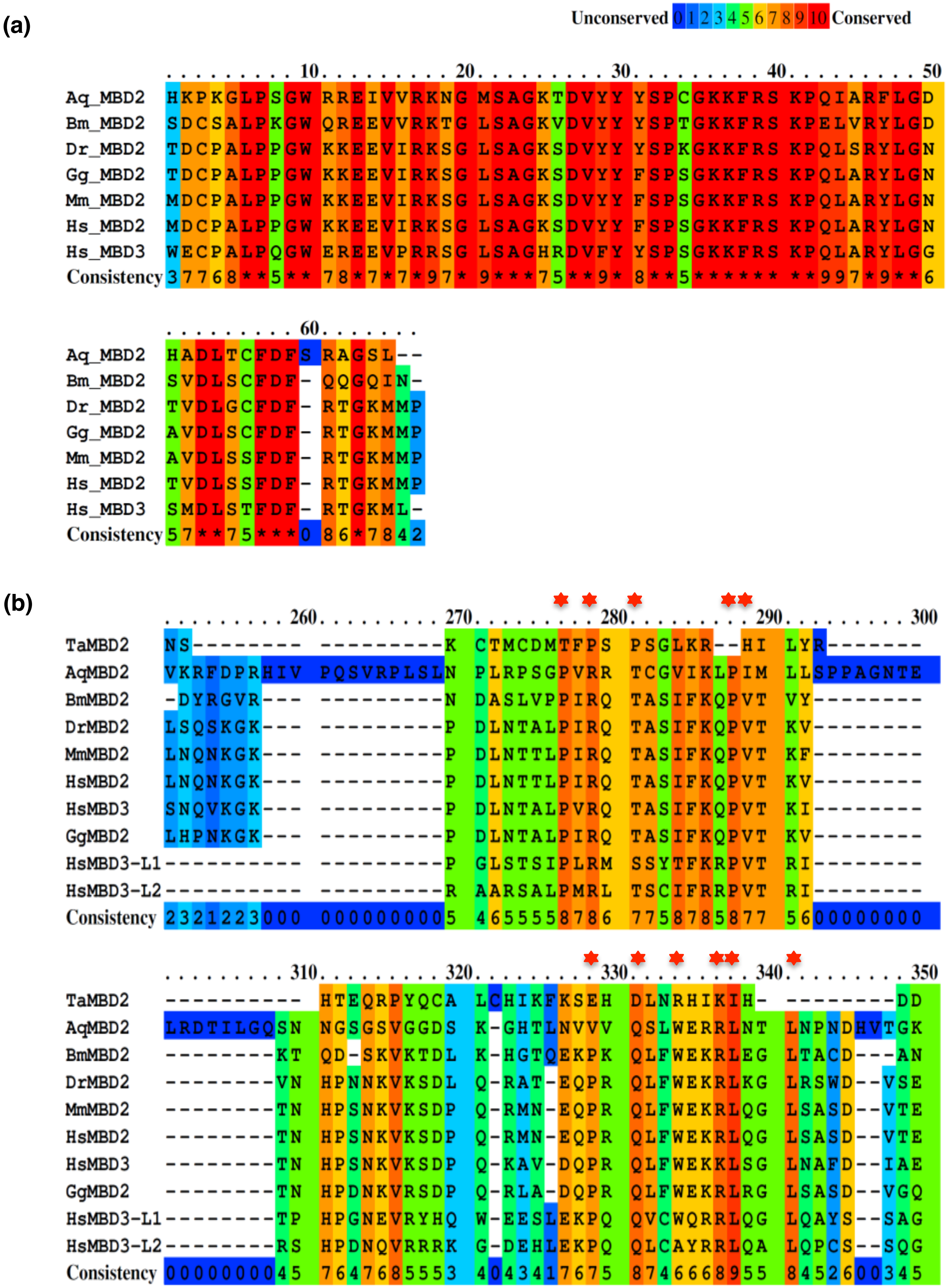

(a)
Unconserved 0 1 2 3 4 5 6 7 8 9 10 Conserved
10 20 30 40 50
Aq_MBD2 HKPKGLPSGW RREIVVRKNG MSAGKTDVYY YSPCGKKFRS KPQIARFLGD
Bm_MBD2 SDCSALPKGW QREEVVRKTG LSAGKVDVYY YSPTGKKFRS KPELVRYLGD
Dr_MBD2 TDCPALPPGW KKEEVIRKSG LSAGKSDVYY YSPKGKKFRS KPQLSRYLGN
Gg_MBD2 TDCPALPPGW KKEEVIRKSG LSAGKSDVYY FSPSGKKFRS KPQLARYLGN
Mm_MBD2 MDCPALPPGW KKEEVIRKSG LSAGKSDVYY FSPSGKKFRS KPQLARYLGN
Hs_MBD2 MDCPALPPGW KKEEVIRKSG LSAGKSDVYY FSPSGKKFRS KPQLARYLGN
Hs_MBD3 WECPALPQGW EREEVPRRSG LSAGHRDVFY YSPSGKKFRS KPQLARYLGG
Consistency 37768**5** 78*7*7*97* 9***75**9* 8**5****** **997*9**6
60
Aq_MBD2 HADLTCFDFS RAGSL--
Bm_MBD2 SVDLSCFDF- QQGQIN-
Dr_MBD2 TVDLGCFDF- RTGKMMP
Gg_MBD2 AVDLSCFDF- RTGKMMP
Mm_MBD2 AVDLSSFDF- RTGKMMP
Hs_MBD2 TVDLSSFDF- RTGKMMP
Hs_MBD3 SMDLSTFDF- RTGKML-
Consistency 57**75***0 86*7842
(b)
260 270 280 290 300
TaMBD2 NS-------- ---------K CTMCDMTFPS PSGLKR--HI LYR-------
AqMBD2 VKRFDPRHIV PQSVRPLSLN PLRPSGPVRR TCGVIKLPIM LLSPPAGNTE
BmMBD2 -DYRGVR--- ---------N DASLVPPIRQ TASIFKQPVT VY--------
DrMBD2 LSQSKGK--- ---------P DLNTALPIRQ TASIFKQPVT KV--------
MmMBD2 LNQNKGK--- ---------P DLNTTLPIRQ TASIFKQPVT KF--------
HsMBD2 LNQNKGK--- ---------P DLNTTLPIRQ TASIFKQPVT KV--------
HsMBD3 SNQVKGK--- ---------P DLNTALPVRQ TASIFKQPVT KI--------
GgMBD2 LHPNKGK--- ---------P DLNTALPIRQ TASIFKQPVT KV--------
HsMBD3-L1 ---------- ---------P GLSTSIPLRM SSYTFKRPVT RI--------
HsMBD3-L2 ---------- ---------R AARSALPMRL TSCIFRRPVT RI--------
Consistency 2321223000 0000000005 4655558786 7758785877 5600000000
310 320 330 340 350
TaMBD2 ---------- HTEQRPYQCA LCHIKFKSEH DLNRHIKIH- --------DD
AqMBD2 LRDTILGQSN NGSGSVGGDS K-GHTLNVVV QSLWERRLNT LNPNDHVTGK
BmMBD2 --------KT QD-SKVKTDL K-HGTQEKPK QLFWEKRLEG LTACD---AN
DrMBD2 --------VN HPNNKVKSDL Q-RAT-EQPR QLFWEKRLKG LRSWD--VSE
MmMBD2 --------TN HPSNKVKSDP Q-RMN-EQPR QLFWEKRLQG LSASD--VTE
HsMBD2 --------TN HPSNKVKSDP Q-RMN-EQPR QLFWEKRLQG LSASD--VTE
HsMBD3 --------TN HPSNKVKSDP Q-KAV-DQPR QLFWEKKLSG LNAFD--IAE
GgMBD2 --------TN HPDNKVRSDP Q-RLA-DQPR QLFWEKRLRG LSASD--VGQ
HsMBD3-L1 --------TP HPGNEVRYHQ W-EESLEKPQ QVCWQRRLQG LQAYS--SAG
HsMBD3-L2 --------RS HPDNQVRRRK G-DEHLEKPQ QLCAYRRLQA LQPCS--SQG
Consistency 0000000045 7647685553 4043417675 8746668955 8452600345

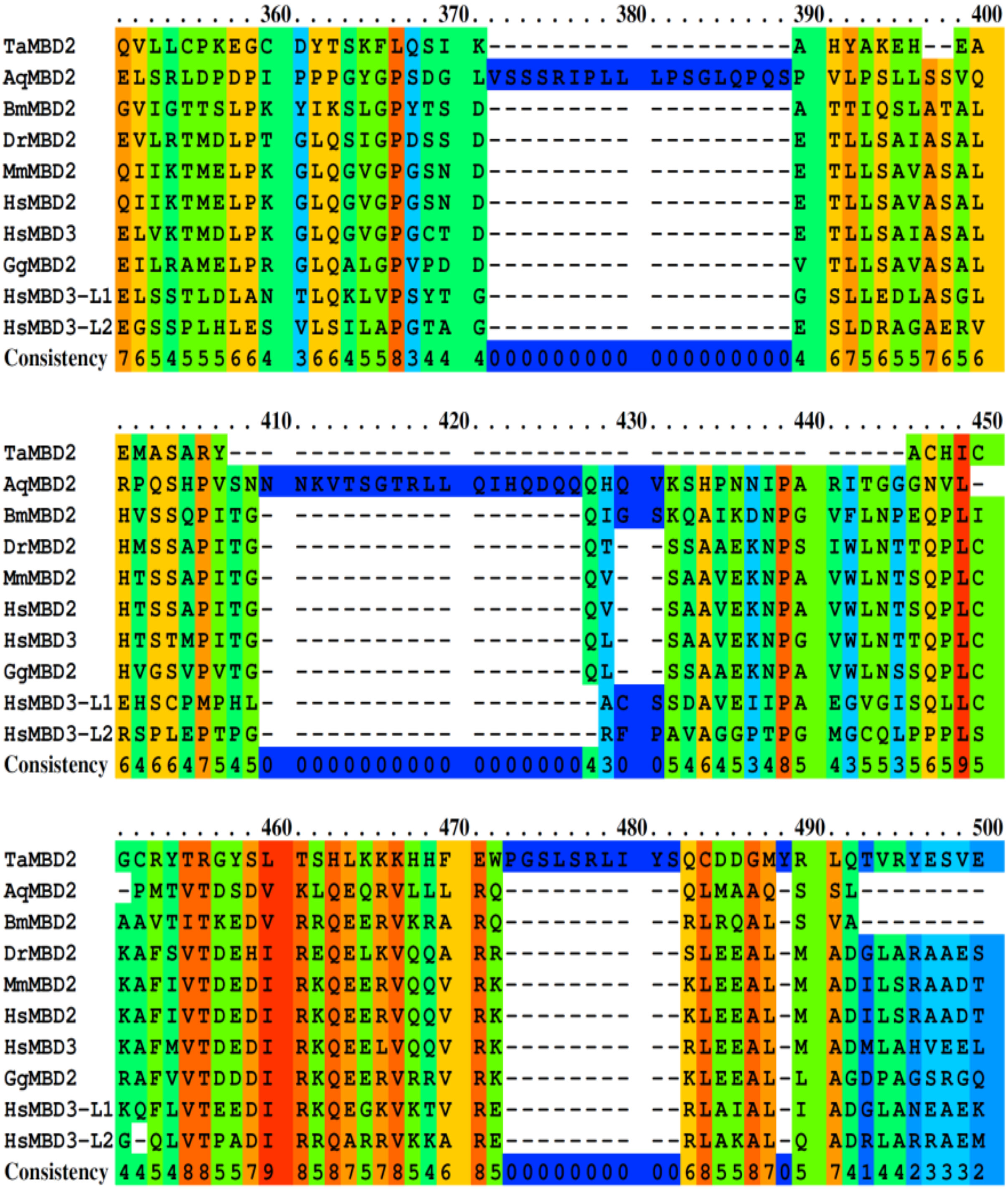
.........360.........370.........380.........390.........400
TaMBD2 QVLLCPKEGC DYTSKFLQSI K--------- ---------A HYAKEH--EA
AqMBD2 ELSRLDPDPI PPPGYGPSDG LVSSSRIPLL LPSGLQPQSP VLPSLLSSVQ
BmMBD2 GVIGTTSLPK YIKSLGPYTS D--------- ---------A TTIQSLATAL
DrMBD2 EVLRTMDLPT GLQSIGPDSS D--------- ---------E TLLSAIASAL
MmMBD2 QIIKTMELPK GLQGVGPGSN D--------- ---------E TLLSAVASAL
HsMBD2 QIIKTMELPK GLQGVGPGSN D--------- ---------E TLLSAVASAL
HsMBD3 ELVKTMDLPK GLQGVGPGCT D--------- ---------E TLLSAIASAL
GgMBD2 EILRAMELPR GLQALGPVPD D--------- ---------V TLLSAVASAL
HsMBD3-L1 ELSSTLDLAN TLQKLVPSYT G--------- ---------G SLLEDLASGL
HsMBD3-L2 EGSSPLHLES VLSILAPGTA G--------- ---------E SLDRAGAERV
Consistency 7654555664 3664558344 4000000000 0000000004 6756557656
.........410.........420.........430.........440.........450
TaMBD2 EMASARY--- ---------- ---------- ---------- -----ACHIC
AqMBD2 RPQSHPVSNN NKVTSGTRLL QIHQDQQQHQ VKSHPNNIPA RITGGGNVL-
BmMBD2 HVSSQPITG- ---------- -------QIG SKQAIKDNPG VFLNPEQPLI
DrMBD2 HMSSAPITG- ---------- -------QT- -SSAAEKNPS IWLNTTQPLC
MmMBD2 HTSSAPITG- ---------- -------QV- -SAAVEKNPA VWLNTSQPLC
HsMBD2 HTSSAPITG- ---------- -------QV- -SAAVEKNPA VWLNTSQPLC
HsMBD3 HTSTMPITG- ---------- -------QL- -SAAVEKNPG VWLNTTQPLC
GgMBD2 HVGSVPVTG- ---------- -------QL- -SSAAEKNPA VWLNSSQPLC
HsMBD3-L1 EHSCPMPHL- ---------- --------AC SSDAVEIIPA EGVGISQLLC
HsMBD3-L2 RSPLEPTPG- ---------- --------RF PAVAGGPTPG MGCQLPPPLS
Consistency 6466475450 0000000000 0000000430 0546453485 4355356595
.........460.........470.........480.........490.........500
TaMBD2 GCRYTRGYSL TSHLKKKHHF EWPGSLSRLI YSQCDDGMYR LQTVRYESVE
AqMBD2 -PMTVTDSDV KLQEQRVLLL RQ-------- --QLMAAQ-S SL--------
BmMBD2 AAVTITKEDV RRQEERVKRA RQ-------- --RLRQAL-S VA--------
DrMBD2 KAFSVTDEHI REQELKVQQA RR-------- --SLEEAL-M ADGLARAAES
MmMBD2 KAFIVTDEDI RKQEERVQQV RK-------- --KLEEAL-M ADILSRAADT
HsMBD2 KAFIVTDEDI RKQEERVQQV RK-------- --KLEEAL-M ADILSRAADT
HsMBD3 KAFMVTDEDI RKQEELVQQV RK-------- --RLEEAL-M ADMLAHVEEL
GgMBD2 RAFVVTDDDI RKQEERVRRV RK-------- --KLEEAL-L AGDPAGSRGQ
HsMBD3-L1 KQFLVTEEDI RKQEGKVKTV RE-------- --RLAIAL-I ADGLANEAEK
HsMBD3-L2 G-QLVTPADI RRQARRVKKA RE-------- --RLAKAL-Q ADRLARRAEM
Consistency 4454885579 8587578546 8500000000 0068558705 7414423332

Figure S3

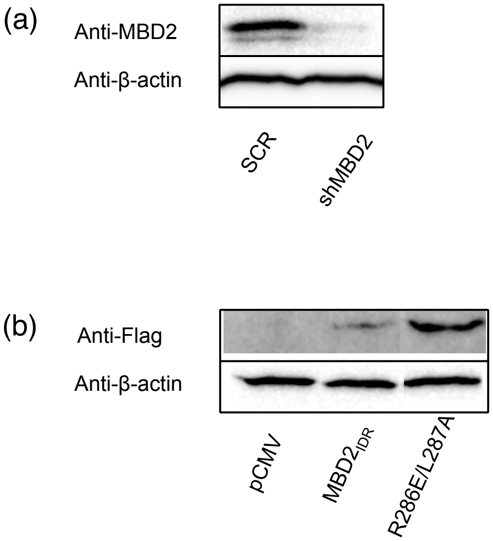
(a)
Anti-MBD2
Anti-β-actin
SCR
shMBD2
(b)
Anti-Flag
Anti-β-actin
pCMV
MBD2IDR
R286E/L287A

Figure S4
